# Evaluation of MicroScan and VITEK 2 systems for susceptibility testing of Enterobacterales with updated breakpoints

**DOI:** 10.1128/jcm.00048-25

**Published:** 2025-04-30

**Authors:** Sandra S. Richter, Elizabeth L. Dominguez, Aaron A. Hupp, Matt Griffis, Shawn H. MacVane

**Affiliations:** 1 Department of Laboratory Medicine and Pathology, Mayo Clinic, Jacksonville, Florida, USA; 2 bioMerieux, Inc. 355122 , Hazelwood, Missouri, USA; Johns Hopkins University, Baltimore, Maryland, USA

**Keywords:** antimicrobial agents, susceptibility testing

## Abstract

We compared the performance of two commercial antimicrobial susceptibility testing (AST) systems for contemporary Enterobacterales strains using broth microdilution (BMD) as the reference standard with 2022 Clinical and Laboratory Standards Institute or current FDA breakpoints applied. Enterobacterales clinical isolates with BMD results were tested in parallel using VITEK 2 AST test cards (N802, XN15, bioMérieux, Hazelwood, MO, USA) and MicroScan NM56 AST panels (Beckman Coulter, Sacramento, CA, USA). The 200 isolates (57 *Escherichia coli*, 55 *Klebsiella pneumoniae*, 21 *Enterobacter cloacae* complex, 18 *Serratia marcescens*, 12 *Proteus mirabilis*, 10 *Citrobacter koseri*, 9 *Citrobacter freundii*, 8 *Klebsiella oxytoca*, 5 *Klebsiella aerogenes*, 3 *Providencia stuartii*, and 2 *Morganella morganii*) included 25% extended-spectrum beta-lactamase (ESBL), 23% carbapenem-resistant Enterobacterales (CRE), and 4.5% AmpC resistance phenotypes. For the 28 antimicrobial agents tested, essential agreement (EA, MIC within ±1 doubling dilution), categorical agreement (CA, same categorical interpretation: susceptible, intermediate, susceptible dose dependent, and resistant), and error rates were calculated using BMD as the reference standard. Time to results (TTR) was determined for each instrument for all 200 isolates. Hands-on time was assessed by timing two technologists each setting up six batches of five isolates on each system. Accuracy was similar between systems with an overall CA > 94% and EA ≥ 96%. The CA was ≥90% for most agents tested on both systems (exceptions were ampicillin-sulbactam, cefoxitin, minocycline, and nitrofurantoin). The MicroScan with Prompt inoculum preparation required less hands-on setup time than VITEK (1.29 vs 1.83 min/isolate). The median instrument TTR was less for VITEK (11.7 vs 18 hours, *P* < 0.001), yielding an overall faster turnaround time.

**IMPORTANCE:**

There are limited data directly comparing the performance of commercial antimicrobial susceptibility testing systems for contemporary bacterial strains using the Clinical and Laboratory Standards Institute broth microdilution method as the reference standard and applying updated breakpoints. These data will hopefully encourage labs to perform the necessary verification or validation studies needed to implement current breakpoints and ensure antimicrobial resistance is detected.

## INTRODUCTION

Reasons for adjustments in the breakpoints used to interpret antimicrobial susceptibility testing (AST) include the emergence of antimicrobial resistance, new pharmacokinetic/pharmacodynamic analyses, and changes in clinical practice (1). The Clinical and Laboratory Standards Institute (CLSI) has a multidisciplinary, consensus process to assess and update breakpoints, but implementation of revised breakpoints by clinical laboratories has been delayed for multiple reasons (1). If the dilutions required for a new breakpoint are available on a laboratory’s commercial AST system panel, then a validation study as outlined in the CLSI M52 document can be performed to enable reporting results with the new breakpoints (2). However, the preferred approach for US laboratories is to utilize tests with Food and Drug Administration (FDA) clearance for reporting the updated breakpoints.

Manufacturers of AST devices are required to apply FDA breakpoints, and a timely process for FDA breakpoint changes to occur was not initially available (3). Initiatives to streamline the FDA’s recognition of updated breakpoints have been supported by CLSI rationale documents (https://clsi.org/meetings/ast/rationale-documents) and an ongoing collaboration between the two organizations. The publication of FDA breakpoints on the Antibacterial Susceptibility Test Interpretive Criteria website and notice of FDA updates are extremely helpful tools (4). Effective January 2024, US laboratories accredited by the College of American Pathologists must implement new breakpoints no later than 3 years after publication by the FDA (MIC.11385) (5).

There are limited data directly comparing the performance of commercial AST systems for contemporary bacterial strains using the CLSI broth microdilution method (BMD) as the reference standard and applying updated breakpoints (6). We examined the accuracy of two commercial AST systems (VITEK 2 and MicroScan) by testing a well-characterized collection of Enterobacterales isolates with BMD as the reference standard. We also compared the workflow (labor and time required to generate results) for each system.

## MATERIALS AND METHODS

### Enterobacterales strains

A collection of 200 Enterobacterales stock isolates frozen in 10% glycerol with CLSI BMD and β-lactamase resistance gene analyses performed by JMI laboratories (Coralville, IA, USA) was sent to the Mayo Clinic microbiology laboratory in Jacksonville, FL, USA, and stored at −70°C (6, 7). The isolates were a subset of those obtained by JMI from US and Latin American medical centers enrolled in their 2015–2019 SENTRY Antimicrobial Surveillance Program or the CDC/FDA Antimicrobial Resistant Isolate Bank (8). The 200 isolates (57 *Escherichia coli*, 55 *Klebsiella pneumoniae*, 21 *Enterobacter cloacae* complex, 18 *Serratia marcescens*, 12 *Proteus mirabilis*, 10 *Citrobacter koseri*, 9 *Citrobacter freundii*, 8 *Klebsiella oxytoca*, 5 *Klebsiella aerogenes*, 3 *Providencia stuartii*, and 2 *Morganella morganii*) included a variety of resistance phenotypes (24.5% ESBL, 24% carbapenem resistant, 4.5% AmpC, and 47% wild type) as outlined in Table 1.

**TABLE 1:** Resistance genotypes of 200 Enterobacterales isolates included in the study

Organism	No. isolates	Wild type	Carbapenemase							ESBL				AmpC
			KPC-2	KPC-3	SME-2	Other serine	MBL	Oxa-48-like	Double	CTX-M-15	CTX-M-27	SHV	Other	
*Citrobacter freundii*	9	4	2	1			1			1				
*Citrobacter koseri*	10	8									1		1	
*Enterobacter cloacae* complex	21	7	1	2		2	3			2		4		
*Escherichia coli*	57	18	2	2			3			13	11	1	2	5
*Klebsiella aerogenes*	5	4	1											
*Klebsiella oxytoca*	8	4		1						2				1
*Klebsiella pneumoniae*	55	23	5	5			3	1	2	6		5	2	3
*Morganella morganii*	2	2												
*Proteus mirabilis*	12	12												
*Providencia stuartii*	3	3												
*Serratia marcescens*	18	9	1	2	2	3	1							
Total	200	94	12	13	2	5	11	1	2	24	12	10	5	9

### Susceptibility testing

The isolates were subcultured twice before being tested in parallel on the VITEK 2 (bioMérieux, Hazelwood, MO, USA) and MicroScan AST systems (Beckman Coulter, Sacramento, CA, USA). Testing was performed using VITEK 2 N802 and XN15 AST cards (9.02 software) and the MicroScan NM56 panel (LabPro 4.42 software) according to the manufacturers’ instructions. The Prompt system was used for the inoculation of the MicroScan panels. Quality control was performed as recommended by each manufacturer. Antimicrobials tested included amikacin, amoxicillin-clavulanic acid, ampicillin, ampicillin-sulbactam, aztreonam, cefazolin, cefepime, cefotaxime, cefoxitin, ceftazidime, ceftazidime-avibactam, ceftolozane-tazobactam, ceftriaxone, cefuroxime, ciprofloxacin, ertapenem, gentamicin, imipenem, levofloxacin, meropenem, meropenem-vaborbactam, minocycline, nitrofurantoin, piperacillin-tazobactam, tetracycline, tigecycline, tobramycin, and trimethoprim-sulfamethoxazole.

### Data analysis

The breakpoints published by CLSI in the February 2022 edition of the M100 and those programmed in the VITEK and MicroScan AST device software reflecting FDA clearance are shown in Table 2 (9). The lower breakpoints for aminoglycosides (amikacin, gentamicin, and tobramycin) published by CLSI in 2023 have not been recognized by the FDA and were not assessed in this study (10). The lower dilutions required for the cefazolin parenteral breakpoints were not available on both panels to allow a comparison. The CLSI cefazolin urine breakpoint to predict the activity of oral agents for uncomplicated urinary tract infections was applied, but it is not recognized by the FDA. Cefotaxime results were not analyzed for MicroScan because the lowest dilution reported (≤2 µg/mL) does not allow the CLSI susceptible breakpoint (≤1 µg/mL) to be applied. Essential agreement (EA, portion of isolates with an MIC result from the commercial device within ±1 doubling dilution of the reference BMD MIC), categorical agreement (CA, portion of isolates with a categorical interpretation [susceptible, intermediate, susceptible dose dependent, and resistant] matching the reference BMD categorical interpretation), and error rates were calculated for each system using BMD as the reference standard.

**TABLE 2:** Comparison of CLSI 2022 breakpoints to the FDA breakpoints programmed in the AST device software^*a*^

	Vitek 2 version 9.02				MicroScan LabPro version 4.42			CLSI M100-ED32, 2022			
Antimicrobial Agent	S	Susceptible dose dependent (SDD)	I	R	S	I	R	S	SDD	I	R
Amikacin	≤16		32	≥64	≤16	32	≥64	≤16		32	≥64
Amoxicillin-clavulanic acid	≤8		16	≥32	≤8	16	≥32	≤8		16	≥32
Ampicillin	≤8		16	≥32	≤8	16	≥32	≤8		16	≥32
Ampicillin-sulbactam	≤8		16	≥32	≤8	16	≥32	≤8		16	≥32
Aztreonam	**≤8**		**16**	**≥32**	**≤8**	**16**	**≥32**	≤4		8	≥16
Cefazolin^b^	≤16		**32**	**≥64**	**≤8**	**16**	≥32	UTI ≤ 16			≥32
Cefepime	≤2		4–8	≥16	**≤8**	**16**	**≥32**	≤2	4–8		≥16
Cefotaxime	≤1		2	≥4	**≤8**	**16–32**	**≥64**	≤1		2	≥4
Cefoxitin	≤8		16	≥32	≤8	16	≥32	≤8		16	≥32
Ceftazidime	**≤8**		**16**	**≥32**	**≤8**	**16**	**≥32**	≤4		8	≥16
Ceftazidime-avibactam	≤8		–	≥16	≤8	–	≥16	≤8			≥16
Ceftolozane-tazobactam	≤2		4	≥8	≤2	4	≥8	≤2		4	≥8
Ceftriaxone	≤1		2	≥4	≤1	2	≥4	≤1		2	≥4
Cefuroxime	≤8		16	≥32	≤8	16	≥32	≤8		16	≥32
Ciprofloxacin	**≤1**		**2**	**≥4**	**≤1**	**2**	**≥4**	≤0.25		0.5	≥1
Ertapenem	≤0.5		1	≥2	≤0.5	1	≥2	≤0.5		1	≥2
Gentamicin	≤4		8	≥16	≤4	8	≥16	≤4		8	≥16
Imipenem	≤1		2	≥4	≤1	2	≥4	≤1		2	≥4
Levofloxacin	**≤2**		**4**	**≥8**	**≤2**	**4**	**≥8**	≤0.5		1	≥2
Meropenem	**≤4**		**8**	**≥16**	**≤4**	**8**	**≥16**	≤1		2	≥4
Meropenem-vaborbactam	≤4		8	≥16	≤4	8	≥16	≤4		8	≥16
Minocycline	≤4		8	≥16	≤4	8	≥16	≤4		8	≥16
Moxifloxacin	≤2		4	≥8	≤2	4	≥8	No CLSI Breakpoints			
Nitrofurantoin	≤32		64	≥128	≤32	64	≥128	≤32		64	≥128
Piperacillin-tazobactam	**≤16**		**32–64**	**≥128**	**≤16**	**32–64**	**≥128**	≤8	16		≥32
Tetracycline	≤4		8	≥16	≤4	8	≥16	≤4		8	≥16
Tigecycline	≤2		4	≥8	≤2	4	≥8	No CLSI Breakpoints			
Tobramycin	≤4		8	≥16	≤4	8	≥16	≤4		8	≥16
Trimethoprim/sulfamethoxazole	≤40		–	≥80	≤40	–	≥80	≤2/38			≥4/76

^*a*^ Boldface values highlight differences between device software and CLSI breakpoints.

^*b*^ Lower dilutions required for CLSI cefazolin parenteral breakpoints were not available on the VITEK cards evaluated.

Isolates with a very major error (VME, false susceptible) or major error (ME, false resistance) were repeated on the VITEK 2 and MicroScan. If the result changed with the second testing, then a third testing was performed on VITEK 2 and MicroScan to obtain a majority result for each AST system. Isolates with a ME or VME confirmed on one of the AST devices had BMD repeated in duplicate at JMI Laboratories using isolates pulled from their stock collection. Any change in the BMD result reflected the majority of all three results (original + duplicate repeat BMD).

### Workflow assessment

Requirements for time to results (TTRs) were determined for each instrument for all 200 isolates. Hands-on time was assessed by timing two different technologists setting up 12 batches of five isolates on each system (six batches per technologist). Daily and weekly maintenance times were assessed with four measurements (two maintenance sessions per technologist).

## RESULTS

The performance of the VITEK 2 and MicroScan systems compared to BMD after error resolution is summarized in Table 3 (initial results are in Table S1). The initial overall EA/CA increased from 95.8%/94.4% to 96.0%/94.5% for VITEK 2 and from 97.3%/94.2% to 98.0%/94.7% for MicroScan after error resolution. Details regarding the final VMEs and MEs are provided in Tables 4 (VITEK) and 5 (MicroScan). The impact of device limitations on the number of results available for each drug is outlined in Table S2. Both systems apply intrinsic resistance rules noted as software-based corrections in the tables. The VITEK 2 expert system compares multiple drugs to a database of phenotypes and applies a yellow flag if there is a correction that will improve the match (8). For most drugs, performance was acceptable and similar on the two systems, fulfilling criteria typically expected for FDA clearance. The CA with 2022 CLSI breakpoints was ≥97% for amikacin, cefazolin/urine, ceftazidime-avibactam, ceftolozane-tazobactam, ceftriaxone, ertapenem, meropenem, meropenem-vaborbactam, and trimethoprim-sulfamethoxazole; ≥93% for ampicillin, cefuroxime, gentamicin, imipenem, piperacillin-tazobactam, tetracycline; and ≥90% for amoxicillin-clavulanic acid, aztreonam, cefepime, ceftazidime, ciprofloxacin, levofloxacin, tigecycline, and tobramycin on both systems. Cefotaxime was only assessed for VITEK 2 (CA 99.5%) due to the limited dilutions available on the MicroScan panel.

**TABLE 3:** Performance of VITEK 2 and MicroScan compared to BMD for 200 isolates of Enterobacterales using CLSI breakpoints after error resolution^*c*^

	Reference broth microdilution (no.)			Vitek 2					MicroScan				
Antimicrobial agent	R	S	I or susceptible dose dependent	EA (%)	CA (%)	VME (no.)	ME (no.)	mE (no.)	EA (%)	CA (%)	VME (no.)	ME (no.)	mE (no.)
Amikacin	6	190	4	97	97.5	0	0	5	100	99	0	0	2
Amoxicillin/clavulanic acid	96	92	12	93.9	91.9	1	0	15	97	93	2	0	12
Ampicillin	158	33	9	98.5	94.2	0	0	8	98.5	94.5	0	0	11
Ampicillin/sulbactam	100	78	22	100	92.7	0	0	10	95.5	82.5	2	0	33
Aztreonam	85	99	16	95	90.5	4	0	15	96.5	92.5	1	0	14
Cefazolin^*a*^	131	69	0	99	99	1	1	NA	99	100	0	0	NA
Cefepime	72	114	14	84.3	90.4	0	0	19	91	91	0	1	17
Cefotaxime^*b*^	101	98	1	97	99.5	0	0	1	NA	NA	NA	NA	NA
Cefoxitin	77	108	15	94.5	87.5	1	0	24	97.5	89.5	1	0	20
Ceftazidime	87	105	8	93	91.5	5	0	12	92	90	1	3	16
Ceftazidime/avibactam	13	187	0	94	100	0	0	NA	100	100	0	0	NA
Ceftolozane/tazobactam	47	151	2	93.9	97.8	0	1	3	99.5	98.5	0	0	3
Ceftriaxone	101	99	0	97.2	99.4	0	1	0	96.5	97.5	0	1	4
Cefuroxime	115	77	8	96	94	0	0	12	98	94.5	0	0	11
Ciprofloxacin	82	111	7	94	91.5	0	0	17	99.5	97	0	0	6
Ertapenem	53	147	0	99.5	99.5	0	0	1	100	100	0	0	0
Gentamicin	37	158	5	99.5	97	0	0	6	99.5	96.5	0	0	7
Imipenem	55	136	9	98.1	97.5	1	0	3	98.2	95.2	0	0	8
Levofloxacin	74	119	7	91	91.5	0	0	17	99	97.5	0	0	5
Meropenem	52	147	1	95	99.5	0	0	1	98.5	99.5	0	0	1
Meropenem/vaborbactam	13	186	1	98	98.5	0	2	1	99.5	99.5	0	0	1
Minocycline	28	159	13	98	90.5	0	0	19	98	89.5	0	0	21
Nitrofurantoin	77	82	41	98.5	85	0	0	30	99	80.5	1	0	38
Piperacillin/tazobactam	56	134	10	96.7	93.4	0	1	11	96.5	93.5	1	0	12
Tetracycline	72	119	9	98	94	1	0	11	99.5	94.5	0	0	11
Tigecycline	3	189	8	93.5	91.5	0	2	15	100	98.4	0	0	3
Tobramycin	46	144	10	98	90.9	0	0	18	99.5	95	0	0	10
Trimethoprim/sulfamethoxazole	72	128	0	100	99.5	1	0	NA	97.5	99	2	0	NA
Total	1,909	3,459	232	96	94.5	15	8	274	98	94.7	11	5	266

^*a*^ Urine breakpoints were applied for cefazolin.

^*b*^ Dilution required to apply cefotaxime susceptible breakpoint was not available on the MicroScan panel.

^*c*^ NA, not applicable (minor errors require the breakpoints to include an intermediate category which these drugs lack)

**TABLE 4:** Categorical (VME and ME) errors between VITEK 2 and BMD using CLSI breakpoints – after error resolution

Antibiotic	Error (no.)	Organism (no. β-lactamase resistance genotype)	Vitek 2 mic	Reference MIC	Software based correction
Amoxicillin/clavulanic acid	VME (1)	*Citrobacter freundii* complex (one wildtype)	≤ 2	32	Yes (intrinsic resistance)
Aztreonam^*a*^	VME (4)	*Escherichia coli* (2 CTX-M-27, 1 CTX-M-15) *Escherichia coli* (1 CTX-M-27)	4 4	16 > 16	No No
Cefazolin^*a*^^,*b*^	VME (1) ME (1)	*Escherichia coli* (one other ESBL) *Citrobacter koseri* (one other ESBL)	8 ≥ 64	>32 16	Yes (phenotype) No
Cefoxitin	VME (1)	*Klebsiella oxytoca* (1 KPC-3)	8	>32	No
Ceftazidime^*a*^	VME (5)	*Escherichia coli* (1 CTX-M-27, 1 CTX-M-15, 1 AmpC, 1 KPC-2) *Escherichia coli* (1 CTX-M-15)	4 4	16 32	No No
Ceftolozane/tazobactam	ME (1)	*Citrobacter koseri* (one other ESBL)	16	0.5	No
Ceftriaxone	ME (1)	*Klebsiella pneumoniae* (1 AmpC)	8	1	No
Imipenem	VME (1)	*Enterobacter cloacae* complex (1 SHV ESBL)	1	4	Yes (phenotype)
Meropenem/vaborbactam	ME (2)	*Serratia marcescens* (one metallo-beta-lactamase) *Serratia marcescens* (1 KPC-2)	32 ≥ 64	4 2	No No
Piperacillin/tazobactam^*a*^	ME (1)	*Klebsiella oxytoca* (1 CTX-M-15)	32	8	No
Tetracycline	VME (1)	*Serratia marcescens* (one wildtype)	4	>16	No
Tigecycline^*c*^	ME (2)	*Enterobacter cloacae* complex (1 KPC-3) *Klebsiella pneumoniae* (one double carbapenemase)	≥8 ≥ 8	2 1	No No
Trimethoprim/sulfamethoxazole	VME (1)	*Serratia marcescens* (1 KPC-2)	40	80	No

^*a*^ Unclaimed performance (the manufacturer does not have FDA clearance to report the results with CLSI breakpoints).

^*b*^ Urine CLSI breakpoints applied (≤16 S; ≥32 R).

^*c*^ No CLSI breakpoints, FDA breakpoints applied.

**TABLE 5:** Categorical (VME and ME) errors between MicroScan and BMD using CLSI breakpoints – after error resolution

Antibiotic	Error	Organism (no. β-lactamase resistance genotype)	MicroScan MIC	Reference MIC	Software based correction
Amoxicillin/clavulanic acid	VME (2)	*Citrobacter freundii* complex (one wildtype) *Serratia marcescens* (one wildtype)	≤ 8 ≤ 8	32 > 32	Yes (intrinsic resistance) Yes (intrinsic resistance)
Ampicillin/sulbactam	VME (2)	*Enterobacter aerogenes* (one wildtype) *Serratia marcescens* (one wildtype)	8 8	64 32	Yes (intrinsic resistance) Yes (intrinsic resistance)
Aztreonam^*a*^	VME (1)	*Serratia marcescens* (one other serine carbapenemase)	≤ 4	16	No
Cefepime^*a*^	ME (1)	*Escherichia coli* (1 CTX-M-15)	> 16	1	No
Cefoxitin	VME (1)	*Serratia marcescens* (one other serine carbapenemase)	≤ 8	32	Yes (intrinsic resistance)
Ceftazidime^*a*^	VME (1) ME (3)	*Serratia marcescens* (1 KPC-2) *Escherichia coli* (1 CTX-M-27) *Serratia marcescens* (one wildtype) *Serratia marcescens* (one other serine carbapenemase)	4 > 16 16 > 16	16 4 0.12 0.25	No No No No
Ceftriaxone	ME (1)	*Escherichia coli* (one other ESBL)	8	0.5	No
Nitrofurantoin	VME (1)	*Klebsiella pneumoniae* (one wildtype)	≤ 32	>64	No
Piperacillin/tazobactam^*a*^	VME (1)	*Serratia marcescens* (one wildtype)	≤ 8	32	No
Trimethoprim/sulfamethoxazole	VME (2)	*Escherichia coli* (1 SHV ESBL) *Serratia marcescens* (1 KPC-2)	20 ≤ 40	160 80	No No

^*a*^ Unclaimed performance (the manufacturer does not have FDA clearance to report the results with CLSI breakpoints).

Nitrofurantoin was problematic for both systems with CA of 85% and 80.5% for VITEK 2 and MicroScan, respectively (all but one error was minor). The CA for ampicillin-sulbactam was only 82.5% on MicroScan due to 2 VME and 33 minor errors compared to 92.7% CA for VITEK. Although CA was ≥90% for aztreonam and ceftazidime with the lower CLSI breakpoints on both systems, there were four to five VMEs on VITEK for these drugs. The lower cefoxitin CA (87.5% and 89.5% on VITEK and MicroScan, respectively) was due to minor errors with only one VME. The CA for minocycline was 90.5% and 89.5% on VITEK and MicroScan, respectively, and all errors were minor. Essential agreement was ≥91% for all agents on both systems with one exception (84.3% EA for cefepime on VITEK 2).

The individual steps and time requirements to set up 12 batches of five isolates on VITEK 2 and MicroScan are outlined in Tables 6 and 7. MicroScan with Prompt inoculum preparation required less hands-on set up time than VITEK (1.29 vs 1.83 min/isolate). The time required for daily and weekly maintenance extrapolated over a 4 week period was nearly 1 hour longer for MicroScan (Table 8), but the monthly maintenance was not assessed for either system. The median instrument TTR was significantly less for VITEK (11.7 vs 18 hours, *P* < 0.001), resulting in an overall faster turnaround time (Table 9).

**TABLE 6:** VITEK 2 time requirements for individual steps per batch of five isolates (*n* = 12 batches)

		Step	Mean time (±SD) in minutes
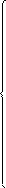 12×	5× 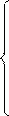	Label tube and purity plate, transfer saline to tube, place in Smart Carrier, add two empty tubes, scan account #, and open/scan/place AST-N802 card in the first empty tube	2.63 ± 0.59
		Inoculate saline tube to 0.5 McFarland, vortex and record McFarland, and open/scan/ place AST-XN15 card in the second empty tube	5.48 ± 1.25
		Place Smart Carrier into VITEK 2	0.20 ± 0.02
		Remove Smart Carrier and streak purity plates	0.85 ± 0.09
Total time per batch of five isolates			9.16 ± 1.37
Total time for 60 isolates			109.95
Total time per isolate			1.83

**TABLE 7:** MicroScan time requirements for individual steps per batch of five isolates (*n* = 12 batches)

	Step	Mean time (±SD) in minutes
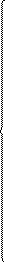 12×	Label five Prompt bottles; for each organism, pick three isolated colonies, place in prompt, and mix; repeat for other four isolates	1.99 ± 0.32
	Open five panel packages; place barcode labels on panels.	1.17 ± 0.11
	Place empty seed tray and associated Prompt bottle by each of the five panels; label purity plates	0.62 ± 0.14
	Pour Prompt bottles in seed trays; streak purity plates for all five isolates	1.55 ± 0.32
	Use Renok device to inoculate the five panels	0.40 ± 0.05
	Place panel cover on each panel; load five panels on the WalkAway instrument	0.75 ± 0.12
Total time per batch of five isolates		6.47 ± 0.53
Total time for 60 isolates		77.62
Total time per isolate		1.29

**TABLE 8:** Maintenance time requirements for VITEK 2 and MicroScan (four measurements)

Maintenance type	VITEK 2	MicroScan
Daily: sum time (4 days) mean time (range)	2 min 35 s 39 s (34–49 s)	9 min 15 s 2 min 19 s (1 min 56 s–2 min 44 s)
Weekly: sum time (4 weeks) mean time (range)	6 min 29 s 1 min 37 s (1 min 14 s–2 min 10 s)	18 min 48 s 4 min 42 s (4 min 29 s–4 min 55 s)
Total/week (daily mean × 7 plus weekly mean)	6 min 10 s	20 min 55 s
Total/month (daily mean × 28 plus weekly sum)	24 min 41 s	83 min 40 s

**TABLE 9:** Time to result in hours for 200 isolates on VITEK 2 and MicroScan WalkAway^a^

Device	Min	Q1	Median	Mean	Q3	Max	SD
VITEK 2	7.73	10.2	11.7	12.7	14.9	18.1	3.13
MicroScan WalkAway	17.1	18.0	18.0	18.1	18.1	18.7	0.216
			Δ −6.36 *P* < 2.2e-16				

^a^ Paired-samples design, difference in median TTR tested with Wilcoxon signed-rank test.

## DISCUSSION

The acceptable performance for most agents observed in our study is encouraging given the new CAP accreditation requirement for laboratories to implement updated breakpoints within 3 years of FDA recognition (https://info.cap.org/antimicrobial-susceptibility-testing/). Reducing the availability of AST system panels and software that allow obsolete breakpoints to be reported will help laboratories become compliant.

In the Fall of 2023, a more limited menu of VITEK 2 Gram-negative AST cards was introduced, which will eventually force US laboratories to stop using cards containing drug formulations available but cleared with obsolete breakpoints. Taking new cards through the FDA clearance process often yields more limitations than the legacy product, which can be frustrating to end users. The additional testing needed to address limitations increases turnaround time for the provision of actionable results and requires laboratories to have multiple AST methods available. With only two exceptions (ceftazidime and cefazolin), the formulations assessed on N802 and XN15 VITEK cards in this study are available on the new cards released in 2023. A new ceftazidime formulation (caz03n, five wells, 0.5–32 µg/mL) replaced caz01n, which should address the elevated VMEs noted in our study with the N802 card. A new cefazolin formulation (cz05n, three wells, 1–32 µg/mL) replaced cz01n on VITEK cards released in 2023, providing the lower dilutions needed for current parenteral breakpoints.

Cefazolin is an example of how a lack of consensus between CLSI and FDA on a breakpoint delays the availability in commercial AST devices. Due to limited dilutions of cefazolin on the N802 card, only CLSI urine breakpoints for oral cephalosporin therapy (susceptible, ≤16 µg/mL; resistant, ≥32) that have not been recognized by the FDA were evaluated for this study (11, 12). The CLSI lowered the parenteral cefazolin susceptible breakpoint for Enterobacterales to ≤1 µg/mL in 2010 based on pharmacokinetic/pharmacodynamic analyses with the FDA-approved dose (1 g every 8 hours). This lower cefazolin breakpoint was recognized by the FDA, but the breakpoint bisects the wild-type population of common Enterobacterales, which impairs test reproducibility, reducing the likelihood that efforts to obtain FDA clearance on an AST device would be successful (1). In 2011, CLSI raised the cefazolin susceptible breakpoint to ≤2 µg/mL based on a frequently prescribed higher dose (2 g every 8 hours), and this breakpoint was recognized by the FDA in October 2021 (4). The VITEK 9.04 software for the 2023 card lists the obsolete FDA cefazolin susceptible breakpoint (≤1 µg/mL). Laboratories can change the breakpoint in the software if their internal validation studies demonstrate adequate performance against a reference standard.

Piperacillin-tazobactam is an essential agent for laboratories to report, but the reliability of commercial AST and disk diffusion methods when testing Enterobacterales has been challenged on multiple occasions (13, 14). Retesting of ESBL-producing isolates collected as part of the Merino trial suggested a potential for false susceptible piperacillin-tazobactam results when testing strains co-harboring CTX-M and OXA-1 enzymes (14, 15). To ensure piperacillin-tazobactam breakpoints for Enterobacterales address the full epidemiology of ESBL-producing strains and acknowledge the role of extended infusion, the 1992 CLSI breakpoints were revised in 2022. This revision moved the MIC of 16 µg/mL, which had been considered susceptible, into a new susceptible dose-dependent (SDD) category, and MICs of 32–64 µg/mL, formerly considered intermediate, were deemed resistant (15). The FDA recently recognized these CLSI breakpoints, but with 16 µg/mL as intermediate rather than SDD (4). Applying the updated CLSI piperacillin-tazobactam breakpoints in our study, the performance was similar for the two systems with 93% CA and predominantly minor errors (5.5% or 6%) plus one ME for VITEK (0.8%) or VME (1.8%) for MicroScan. This was a better performance than reported in a smaller study assessing the updated piperacillin-tazobactam breakpoints for 55 isolates on MicroScan (80% CA, 36.4% VMEs, and 4.8% MEs) and 62 isolates on VITEK 2 (95.2% CA and 6.3% VMEs) (16). The lower number of piperacillin-tazobactam results for VITEK (*n* = 182) in our study was due to a limitation with *Serratia marcescens,* preventing results for this drug from being available.

Nitrofurantoin CA was low on both systems, and CLSI has made no updates to the breakpoints initially established for this drug. Although updating breakpoints is a burden for clinical laboratories and device manufacturers, the process ultimately helps improve device performance. The clinical data supporting nitrofurantoin breakpoints to guide treatment of uncomplicated UTIs are limited (17). This explains why the EUCAST guidance for Enterobacterales restricts the application of nitrofurantoin breakpoints to *E. coli* causing uncomplicated UTIs. Only two of the VITEK and MicroScan nitrofurantoin minor errors in our study occurred for *E. coli,* with the rest spread across multiple species (4 or 6 *C. koseri*, 1 *C. freundii* complex, 7 *E. cloacae* complex, 3 *K. aerogenes*, 2 *K. oxytoca* [VITEK only], 8 or 17 *K. pneumoniae*, 2 or 1 *M. morganii,* and 1 *S. marcescens* [MicroScan only]). Most of the nitrofurantoin minor errors were due to the commercial system undercalling the result (22/30 for VITEK; 28/38 for MicroScan). An elevated minor error rate for nitrofurantoin was reported in an earlier VITEK study (18).

Cefepime error rates were similar for the two systems despite the lower EA observed on VITEK 2 (84.3%) compared to MicroScan (91.0%). The lower range of cefepime MICs that can be reported on VITEK (≤0.12 µg/mL) contributed to the lower EA. Seven of the VITEK cefepime results outside of EA were MICs ≤ 1.0 µg/mL below the MicroScan lowest calling range of ≤2.0 µg/mL, which is the susceptible breakpoint. The 18 MicroScan cefepime errors were all overcalling resistance, with 1 major and 17 minor errors (14 Microscan R; 3 MicroScan SDD). The 19 VITEK cefepime errors were all minor, with seven overcalling resistance (VITEK R) and the other 12 underestimating the categorical interpretation as VITEK S (*n* = 4) or VITEK SDD (*n* = 8). Our study was not designed to explore the inoculum effect phenomenon that has been reported for cefepime when testing multidrug-resistant organisms (19).

Automated AST systems have been adopted by most laboratories because they provide important results in a more clinically relevant time frame with less labor than manual reference methods (20). Expert systems allow analysis of complex results to be assessed in a more critical manner than possible with human review to prevent errors (8). The smaller amount of time required to set up isolates on MicroScan was attributed to using the Prompt system rather than preparing an inoculum to a specific McFarland as required for VITEK 2. The maintenance time differences between systems would likely be reduced if the monthly maintenance had been included in the study. The shorter run time is an advantage of VITEK 2, assuming the AST results are released and acted upon when they become available.

Limitations of our study include the use of previously determined BMD results rather than testing all methods concurrently in parallel. The discordant analysis that included retesting of isolates with reproducible VME and ME at JMI by BMD helps address this weakness. The BMD retesting was performed using the JMI stock collection and would not address potential loss of resistance that could have occurred during subcultures performed at the study site. The workflow assessment was based on a relatively small number of measurements by only two individuals. The 1.83 min VITEK time per isolate in our study was very close to a previous evaluation that reported 1.68 min per isolate (21).

In conclusion, the performance of both commercial AST systems using this challenging collection of Enterobacterales was acceptable for most agents with updated breakpoints when compared to the BMD reference standard. This should encourage laboratories to perform the necessary verification or validation studies needed to implement current breakpoints and ensure antimicrobial resistance is detected.

## SUPPLEMENTAL MATERIAL

Tables S1 and S2 jcm.00048-25-s0001.doc Table S1: Initial performance. Table S2: Device limitations.

ASM does not own the copyrights to Supplemental Material that may be linked to, or accessed through, an article. The authors have granted ASM a non-exclusive, world-wide license to publish the Supplemental Material files. Please contact the corresponding author directly for reuse.
