# Supplementary material for: Evaluation of MicroScan and VITEK 2 systems for susceptibility testing of Enterobacterales with updated breakpoints
Source: J Clin Microbiol. 2025 Apr 30;63(6):e00048-25. doi: 10.1128/jcm.00048-25 (PMC12153288; doi:10.1128/jcm.00048-25)
Supplement: Tables S1 and S2 — Table S1: Initial performance. Table S2: Device limitations. [file jcm.00048-25-s0001.doc]

| Table S1. Initial performance of Vitek 2 and MicroScan for 200 isolates of Enterobacterales using CLSI Breakpoints | | | | | | | | |  |  |  |  |  |  |  |
| --- | --- | --- | --- | --- | --- | --- | --- | --- | --- | --- | --- | --- | --- | --- | --- |
|  | Reference Broth Microdilution (no.) | | |  | Vitek 2 | | | | |  | MicroScan | | | | |
| Antimicrobial agent | R | S | I or SDD |  | EA (%) | CA (%) | VME (no.) | ME (no.) | mE (no.) |  | EA (%) | CA (%) | VME (no.) | ME (no.) | mE (no.) |
| Amikacin | 6 | 190 | 4 |  | 96.4 | 97.5 | 0 | 0 | 5 |  | 99.5 | 98.5 | 0 | 1 | 2 |
| Amoxicillin/clavulanic acid | 96 | 92 | 12 |  | 93.9 | 91.9 | 2 | 0 | 14 |  | 96.5 | 92.5 | 2 | 1 | 12 |
| Ampicillin | 158 | 33 | 9 |  | 98.5 | 94.2 | 0 | 0 | 8 |  | 97.0 | 93.0 | 2 | 1 | 11 |
| Ampicillin/sulbactam | 101 | 78 | 21 |  | 100.0 | 92.7 | 0 | 0 | 10 |  | 93.5 | **81.5** | 4 | 2 | 31 |
| Aztreonam | 85 | 99 | 16 |  | 95.0 | 90.5 | 4 | 0 | 15 |  | 95.0 | 91.0 | 2 | 2 | 14 |
| Cefazolin^a^ | 131 | 69 | 0 |  | 99.0 | 99.0 | 1 | 1 | 0 |  | 99.0 | 99.5 | 0 | 1 | 0 |
| Cefepime | 72 | 115 | 13 |  | **83.8** | **89.9** | 1 | 0 | 19 |  | 91.0 | 91.0 | 0 | 2 | 16 |
| Cefotaxime^b^ | 100 | 99 | 1 |  | 96.5 | 99.0 | 0 | 1 | 1 |  | NA | NA | NA | NA | NA |
| Cefoxitin | 79 | 108 | 13 |  | 94.0 | 87.5 | 3 | 0 | 22 |  | 96.0 | **88.5** | 2 | 2 | 19 |
| Ceftazidime | 89 | 104 | 7 |  | 92.5 | 91.0 | 7 | 0 | 11 |  | 91.0 | 89.0 | 2 | 5 | 15 |
| Ceftazidime/avibactam | 13 | 187 | 0 |  | 94.0 | 100.0 | 0 | 0 | 0 |  | 98.5 | 98.5 | 1 | 2 | 0 |
| Ceftolozane/tazobactam | 47 | 151 | 2 |  | 93.9 | 97.2 | 0 | 2 | 3 |  | 99.5 | 98.5 | 0 | 0 | 3 |
| Ceftriaxone | 101 | 99 | 0 |  | 97.7 | 100.0 | 0 | 0 | 0 |  | 95.5 | 97.0 | 0 | 4 | 2 |
| Cefuroxime | 115 | 77 | 8 |  | 96.0 | 94.0 | 0 | 0 | 12 |  | 97.5 | 94.5 | 0 | 1 | 10 |
| Ciprofloxacin | 82 | 111 | 7 |  | 94.0 | 91.5 | 0 | 0 | 17 |  | 99.0 | 96.5 | 0 | 1 | 6 |
| Ertapenem | 53 | 147 | 0 |  | 99.5 | 99.5 | 0 | 0 | 1 |  | 100.0 | 100.0 | 0 | 0 | 0 |
| Gentamicin | 37 | 158 | 5 |  | 99.5 | 97.0 | 0 | 0 | 6 |  | 98.5 | 95.5 | 0 | 3 | 6 |
| Imipenem | 55 | 136 | 9 |  | 98.1 | 97.5 | 1 | 0 | 3 |  | 97.0 | 94.6 | 0 | 2 | 7 |
| Levofloxacin | 74 | 119 | 7 |  | 91.0 | 91.5 | 0 | 0 | 17 |  | 98.5 | 97.0 | 0 | 1 | 5 |
| Meropenem | 52 | 147 | 1 |  | 95.0 | 99.5 | 0 | 0 | 1 |  | 98.5 | 99.5 | 0 | 0 | 1 |
| Meropenem/vaborbactam | 13 | 186 | 1 |  | 98.0 | 98.5 | 0 | 2 | 1 |  | 99.5 | 100.0 | 0 | 0 | 0 |
| Minocycline | 29 | 159 | 12 |  | 97.0 | 90.0 | 2 | 0 | 18 |  | 97.5 | **89.5** | 1 | 0 | 20 |
| Nitrofurantoin | 78 | 82 | 40 |  | 98.0 | **85.5** | 1 | 0 | 28 |  | 98.5 | **81.0** | 2 | 0 | 36 |
| Piperacillin/tazobactam | 56 | 135 | 9 |  | 96.2 | 93.4 | 0 | 2 | 10 |  | 96.0 | 93.0 | 1 | 1 | 12 |
| Tetracycline | 72 | 119 | 9 |  | 97.5 | 94.0 | 1 | 1 | 10 |  | 99.5 | 95.0 | 0 | 0 | 10 |
| Tigecycline | 4 | 190 | 6 |  | 92.5 | 91.5 | 1 | 3 | 13 |  | 99.5 | 98.4 | 1 | 0 | 2 |
| Tobramycin | 46 | 144 | 10 |  | 98.0 | 90.9 | 0 | 0 | 18 |  | 98.5 | 94.0 | 0 | 1 | 11 |
| Trimethoprim/sulfamethoxazole | 71 | 129 | 0 |  | 99.5 | 99.5 | 1 | 0 | 0 |  | 97.0 | 98.0 | 2 | 2 | 0 |
| Total | 1915 | 3463 | 222 |  | 95.8 | 94.4 | 25 | 12 | 263 |  | 97.3 | 94.2 | 22 | 35 | 251 |

^a^Urine breakpoints were applied for cefazolin.

^b^Dilution required to apply cefotaxime susceptible breakpoint was not available on the MicroScan panel.

| Table S2. Impact of device limitations on number of results available | | |
| --- | --- | --- |
| Antimicrobial agent | Vitek 2 (no. results) | MicroScan (no. results) |
| Amikacin^1^ | 197 | 200 |
| Amoxicillin/clavulanic acid^1^ | 197 | 200 |
| Ampicillin^2^ | 137 | 200 |
| Ampicillin/sulbactam^2^ | 137 | 200 |
| Aztreonam | 200 | 200 |
| Cefazolin | 200 | 200 |
| Cefepime^3^ | 198 | 200 |
| Cefotaxime^4^ | 200 | NA |
| Cefoxitin | 200 | 200 |
| Ceftazidime | 200 | 200 |
| Ceftazidime/avibactam | 200 | 200 |
| Ceftolozane/tazobactam^5^ | 180 | 200 |
| Ceftriaxone^6^ | 177 | 200 |
| Cefuroxime | 200 | 200 |
| Ciprofloxacin | 200 | 200 |
| Ertapenem^7^ | 197 | 200 |
| Gentamicin | 200 | 200 |
| Imipenem^8,9^ | 160 | 167 |
| Levofloxacin | 200 | 200 |
| Meropenem | 200 | 200 |
| Meropenem/vaborbactam | 200 | 200 |
| Minocycline | 200 | 200 |
| Nitrofurantoin | 200 | 200 |
| Piperacillin/tazobactam^10^ | 182 | 200 |
| Tetracycline | 200 | 200 |
| Tigecycline^11^ | 200 | 188 |
| Tobramycin^1^ | 197 | 200 |
| Trimethoprim/sulfamethoxazole | 200 | 200 |
| ^1^ Vitek limitation (no. isolates): Alternate method required for *P. stuartii* (3). | | |
| ^2^ Vitek: Alternate method required for *C. freundii* (9), *C. koseri* (10), *E. cloacae* (21), *K. aerogenes*(5), *S. marcescens* (18). | | |
| ^3^ Vitek: Alternate method required for *M. morganii* (2). | |  |
| ^4^MicroScan: Cefotaxime MIC range does not include dilution required to apply CLSI susceptible breakpoint. | | |
| ^5^ Vitek: Alternate method required for *M. morganii* (2), *S. marcescens* (18). | | |
| ^6^ Vitek: Alternate method required for *E. cloacae* (21), *M. morganii* (2). | |  |
| ^7^ Vitek: Alternate method required for isolates with ertapenem MIC of 0.25 or 0.5 µg/mL. | | |
| ^8^ Vitek: Alternate method required for *K. aerogenes* (5), *P. mirabilis* (12), *P. stuartii* (3), *M. morganii* (2), *S. marcescens* (18). | | |
| ^9^ MicroScan: Alternate method required for *P. mirabilis* (12), *P. stuartii* (3), *S. marcescens* (18). | | |
| ^10^ Vitek: Alternate method required for *S. marcescens* (18). | |  |
| ^11^ MicroScan: Alternate method required for *P. mirabilis* (12). | |  |
